# Dynamical Behavior of Coal Shearer Traction-swing Coupling under Corrected Loads

**DOI:** 10.1038/s41598-020-65184-w

**Published:** 2020-05-25

**Authors:** Xinwei Yang, Hongyue Chen, Jun Mao, Yajing Wei

**Affiliations:** 1 Faculty of Electrical and Control Engineering, Liaoning Technical University, Huludao, Liaoning 125105 China; 2 School of Mechanical Engineering, Liaoning Technical University, Fuxin, Liaoning 123000 China; 3 Fuxin City Industrial Technology Research Institute, Fuxin, Liaonin 123000 China

**Keywords:** Mechanical engineering, Nonlinear phenomena

## Abstract

Vibration is a major concern in coal mining with a shearer, and an accurate model that allows complex responses can analyze the overall vibration of the system. The large load impact on and severe vibration of a coal shearer under operating conditions were considered. A numerical model was proposed for characterizing the nonlinear dynamics of the shearer traction-swing coupling in 13 degrees of freedom using vibration mechanics and multibody dynamics. Particularly, the contact between the shearer sliding shoe and scraper conveyor was characterized using three-dimensional fractal theory, the gapped contact between the driving wheel and base plate was characterized using Hertz contact theory, and the rigidity of the lift cylinder, the coupling between the shearer fuselage and haulage unit, and the rigidity of the shearer ranging arm were characterized using Hooke’s law. Using experimentally corrected drum loads as the external excitation, the numerical model was resolved to characterize and analyze the dynamical responses of critical shearer components. The numerical model was validated against the vibration responses of a shearer and its critical components under different operating conditions obtained from a mechanical test.The research results provide theoretical basis for the structure optimization and process parameter optimization of the shearer.

## Introduction

The most critical machine unit in fully mechanized coal face is the shearer and its reliability directly impacts mining productivity. The reliability and vibration of the shearer and its critical components have been researched. Hoseinie *et al*.^1–4^ analyzed the reliability of shearer traction, cabling, and hydraulic systems using failure data collected from the Tabas Coal Mine in Iran. Their system time-to-failure data satisfied a three-parameter Weibull distribution and helped optimize system maintenance schedules. Liu *et al*.^5^ experimentally obtained the vibration response of the shearer traction mechanism and the influence pattern, providing theoretical criteria for walking mechanism design. Through a quantitative analysis of the dynamical performance of a shearer planetary gear system, Zhou *et al*.^6^ evaluated the failure risk of the system, obtained the probability distribution of the system’s maximum contraction stress by saddle-point approximation, and performed a Monte Carlo simulation of the system’s reliability. Chen *et al*.^7,8^ comprehensively considered the contact characteristics of the shearer and the scraper conveyor. Based on a virtual prototype technology, a roller experimental load was used as the external stimulus, the mechanical characteristics and fatigue life of flat and guidance sliding boots of the shearer were analyzed, and its structure was optimized. Mao *et al*.^9^ took the MG500/1180–WD shearer as their research object and experimentally obtained the dynamic characteristic curve of the shearer cutting units under different conditions, thereby providing experimental data for the dynamic analysis of shearer cutting sections. Jiang *et al*.^10^ established an electromechanical coupling dynamic model of the shearer cutting unit. Based on the Routh–Hurwitz criterion, the Hopf bifurcation characteristics and the type and effects of torsional vibration of the system were determined. Moreover, the influences of linear and nonlinear gain on the bifurcation point and the limit cycle amplitude were discussed and analyzed. Liu *et al*.^11^ analyzed the influence of different forms of cutting teeth on the vibration characteristics of a shearer through experimental methods. Their research results have guiding significance for the selection of shearer cutting teeth in actual industrial production. The optimal design of the cutting teeth structure also has theoretical reference value. Sheng *et al*.^12^ constructed a nonlinear dynamic model of the electromechanical coupling of the shearer cutting unit, based on electric machine theory and the Maxwell equation; the electromagnetic excitation and the load of the drum were taken as the external excitation of the system. The influence of different operating parameters on the vibration characteristics of the system was analyzed. Wang *et al*.^13^, constructed a electromechanical coupling dynamic model of the transmission system of a shearer cutting unit. The dynamic and electrical characteristics of the system were analyzed, and the change laws of the dynamic characteristics of the drum, induction motor, and gear transmission system according to the load were established. Daolong *et al*.^14^ constructed a dynamic model of shearer cutting units in the vertical direction. The harmonic excitation was considered as the external excitation of the system. The influence of the support characteristics of the cylinder on the vibration characteristics of the system was investigated. Zeng *et al*.^15^ simulated and analyzed the dynamic characteristics of shearer cutting units based on finite-element methods and co-simulation technology. The change laws of the meshing force of gears and the vibration acceleration and frequency of the transmission system under different working conditions were obtained. Zhang *et al*.^16^ introduced a vibration state monitoring method of a coal shearer. The vibration signals obtained by this method were analyzed, the rules of the mechanical fault of the shearer were established, and the feasibility of this method was verified. Ge *et al*.^17^ comprehensively considered the dynamic and the drum load characteristics of a driving motor of a shearer cutting unit. An electromechanical coupling dynamic model of the transmission system was established and an active control strategy was proposed to suppress the dynamic characteristics of the transmission system caused by the mutational external load. The control strategy was also validated. Qin *et al*.^18^ constructed a dynamic model of the housing and transmission system based on a finite element method and a dynamic sub-structuring method; they analyzed the impact of optimizing the housing on the dynamic characteristics of the housing and the transmission system.

The limitations of the studies above are they either only analyzed the dynamics of the shearer in individual directions or the dynamics of its individual constituents. As any large machine with a complex structure, the shearer has components that vibrate and interact to form complex behavior. The studies mentioned also did not provide a detailed description of the rigidity of the coupling between shearer components. To address this knowledge gap, the present study tested the feasibility and accuracy of simulation solutions, proposed a coefficient capable of correcting shearer drum load against traction speed, and experimentally determined the coefficient. Our numerical models describe the rigidity of critical shearer components and of the gapped contact between them, with three-dimensional fractal theory, Hertz contact theory, and Hooke’s law. The numerical model serves to characterize the nonlinear dynamics of the shearer traction-swing coupling in 13 degrees of freedom using both vibration mechanics and multi-body dynamics. Vibration responses of critical shearer components under different operating conditions were obtained from experimentally corrected drum load as the external excitation of the shearer system. The study was concluded with the experimental validation of the model’s accuracy to predict the reliability of the shearer and the lifetime of its critical components.

## Numerical and Experimental Analyses of Shearer Drum Load

### Drum load correction

The conventional computation of drum loads is remarkably inconsistent with experimental data. The shearer drum load is an important input to describe shearer dynamics. However, shearer dynamics diverge from experimentally obtained drum loads as the external excitation. Therefore, the conventional formula for drum load^19^ was corrected against experimental results to obtain an accurate solution of shearer dynamics.

The formula for correcting drum load against traction speed was expressed as follows:

1 $$\{\begin{array}{c}{R}_{gx}^{{\rm{{\prime} }}}={k}_{vx}\cdot \mathop{\sum }\limits_{i=1}^{{N}_{c}}({Z}_{i}\,\cos \,{\varphi }_{i}+{Y}_{i}\,\sin \,{\varphi }_{i})\\ {R}_{gy}^{{\rm{{\prime} }}}={k}_{vy}\cdot \mathop{\sum }\limits_{i=1}^{{N}_{c}}(-{Z}_{i}\,\sin \,{\varphi }_{i}+{Y}_{i}\,\cos \,{\varphi }_{i})\\ {R}_{gz}^{{\rm{{\prime} }}}={k}_{vz}\cdot \mathop{\sum }\limits_{i=1}^{{N}_{c}}({X}_{i})\end{array}$$

where *R*_*gx*_, *R*_*gy*_, and *R*_*gz*_ are drum cutting loads, in traction, vertical, and axial directions, respectively; *M*_*g*_ is the drum cutting torque; *N*_*c*_ is the number of drum picks involved in the cutting; *R*_*g*_ is the radius of the drum; *φ*_*i*_ is the angle between the i-th pick and the vertical direction; *X*_*i*_, *Y*_*i*_, and *Z*_*i*_ are i-th resistances in the lateral, traction, and cutting directions, respectively; and *k*_*vx*_, *k*_*vy*_, and *k*_*vz*_ are the coefficients for correcting the drum loads in the x, y, and z directions against traction speed, respectively.

### Drum load test program

A shearer cutting test was performed using the test platform for mechanical inspection and analysis of fully mechanized coal face units at the National Energy Mining Equipment Research and Development Center. This was chosen as field tests have limitations and the data from such tests are not sufficiently reliable. The test was performed on an MG500/1130WD drum shearer.

**Figure 1:**
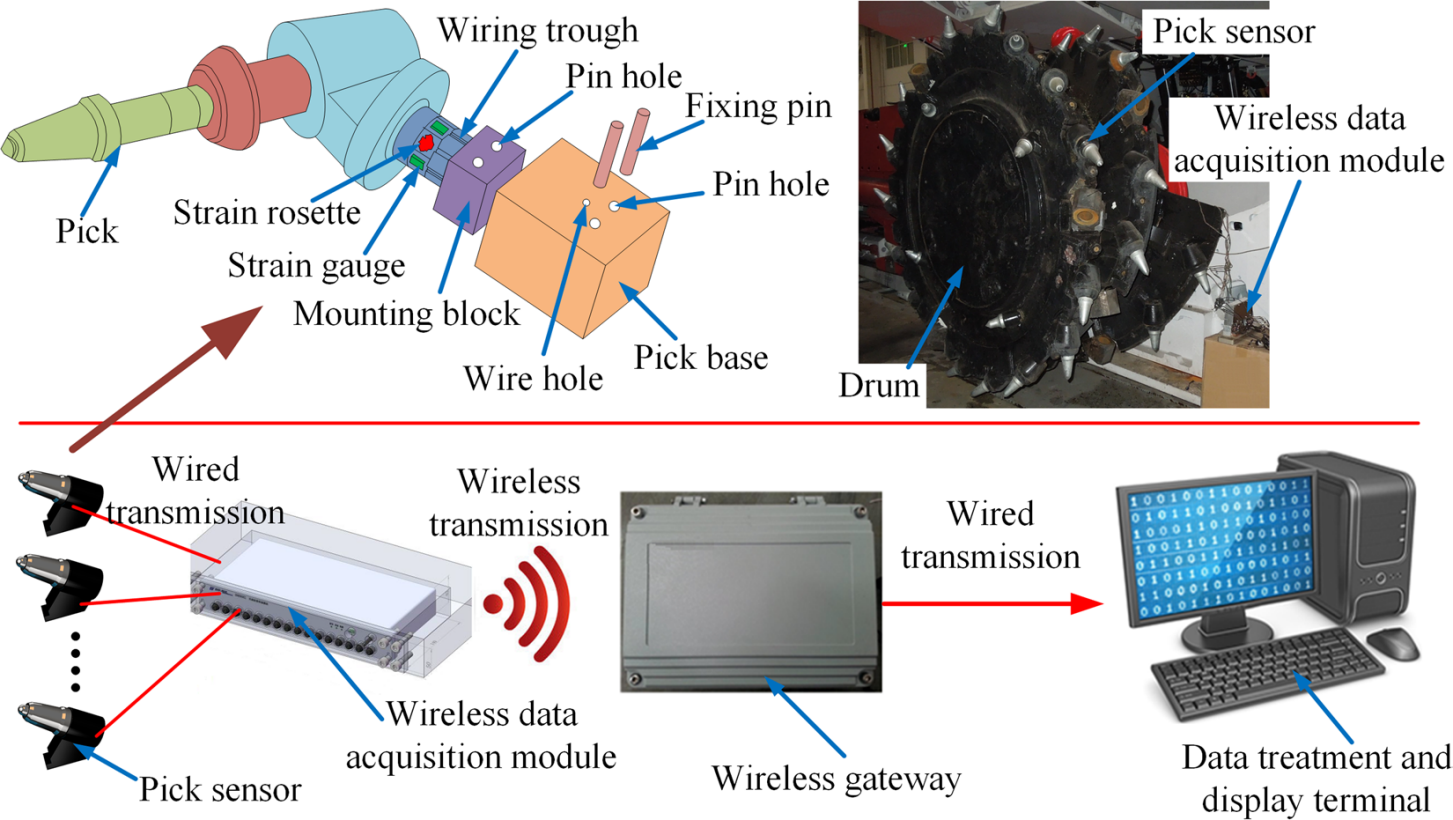
Pick load data acquisition system.

Nine pick sensors were mounted on the drum, as shown in Fig. 1. The leads of the strain gauges and strain rosettes of the sensors were wired through the troughs and guiding holes on the pick base and connected to the wireless data acquisition module mounted at the end of the drum spiral blade assembly. The pick sensor data was transmitted to a data acquisition terminal. After removing noise, the data was then used to compute the loads on the drum picks and then on the drum in the three directions.

The cutting loads on the drum in the three directions at different traction speeds (1.5, 2, 2.5, 3, and 3.5 m/min) were obtained to determine the drum load correction coefficients in Eq. (1). The average cutting loads on the drum in the three directions at different traction speeds are presented in Table 1. Here, the traditional calculation value is determined using the traditional roller load formula, and the testing value is obtained by the roller load experiment.

**Table 1:** Drum cutting loads yielded by the test and the conventional formula.

Operating conditions *v* (m/min)	Traction direction *R*_*gx*_/kN			Vertical direction *R*_*gy*_/kN			Axial direction *R*_*gz*_/kN		
	Testing value results	Traditional calculated value	Ratio of testing value to theoretical value	Testing value results	Traditional calculated value	Ratio of testing value to theoretical value	Testing value results	Traditional calculated value	Ratio of testing value to theoretical value
1.5	33.52	35.43	0.946	20.13	22.51	0.894	7.87	8.84	0.890
2	35.23	36.75	0.958	22.56	24.68	0.914	9.42	10.26	0.918
2.5	41.34	42.52	0.972	30.17	32.82	0.919	17.22	18.78	0.922
3	46.62	47.38	0.978	33.35	36.12	0.923	20.15	21.69	0.929
3.5	51.42	52.45	0.980	40.35	43.18	0.934	27.35	29.28	0.934

The coefficients used to correct drum loads in the three directions against the traction speed were determined as follows:

2 $$\{\begin{array}{c}{k}_{vx}=0.932+0.018\cdot v\\ {k}_{vy}=0.872+0.018\cdot v\\ {k}_{vz}=0.869+0.020\cdot v\end{array}$$

where *v* is the shearer traction speed.

Table 2 compares the corrected traditionally calculated value, and testing values of the drum cutting loads in the three directions at a traction speed of 3 m/min. The corrected simulation values are closer to the testing values, with relative errors of 0.77%, 2.67%, and 1.59% in the three directions.

**Table 2:** Comparison of corrected numerical simulation results and testing results.

Testing conditions	Traction direction *R*_*gx*_/kN			Vertical direction *R*_*gy*_/kN			Axial direction *R*_*gz*_/kN		
	Testing value results	Correction value	Error %	Testing value results	Correction value	Error %	Testing value results	Correction value	Error %
*v* = 3 m/min	46.62	46.98	0.77	33.35	34.23	2.67	20.15	20.47	1.59

## Dynamic Behavior of Shearer Traction-Swing Coupling

### Numerical simulation of traction-swing coupling dynamics

**Figure 2:**
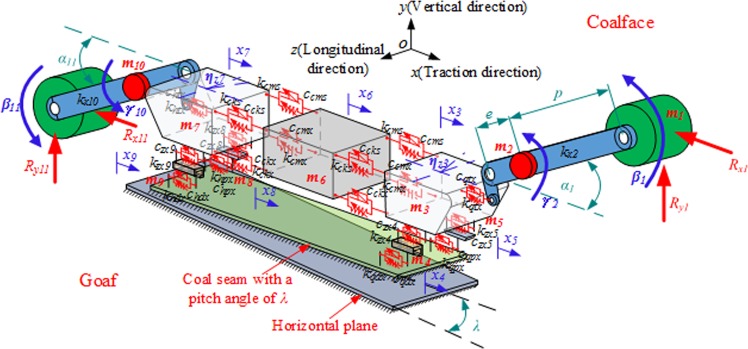
Model of shearer traction-swing coupling nonlinear dynamics.

A shearer has a complex structure. For a better representation of the dynamical behavior of shearer traction-swing coupling, the shearer was divided into the following 11 components using a lumped parameter method: front and rear drums, front and rear ranging arms, front and rear haulage unit, front and rear walking units, front and rear supporting units, and body. Figure 2 shows a model of the shearer traction-swing coupling dynamics. The following assumptions were made:

1. the mass of a shearer component is concentrated at one point–its gravity center;
2. the effect of the dynamics of the shearer hydraulic, electrical, and power transmission systems on the dynamics of the whole shearer is negligible;
3. the mass of the frontal section of the front and rear ranging arms (which are connected to the front and rear drums) is negligible;
4. the shearer is a rigid dynamical system. Its components are simulated as rigid and damping components to describe the contact and coupling between them.

The symbols in the figure are explained in Table 3.

**Table 3:** Explanation of symbols in Fig. 2.

Symbol	Explanation
*m*_*1*_*, m*_*11*_	The masses of the front and rear drums
*m*_*2*_*, m*_*10*_	The masses of the front and rear ranging arms
*m*_*3*_*, m*_*7*_	The masses of the front and rear haulage unit
*m*_*4*_*, m*_*9*_	The masses of the front and rear walking units
*m*_*5*_*, m*_*8*_	The masses of the front and rear supporting units
*m*_*6*_	The mass of the shearer body
*x*_*3*_*, x*_*7*_	The vibration displacements of the front and rear haulage unit in the traction direction
*x*_*4*_*, x*_*9*_	The vibration displacements of the front and rear walking units in the traction direction
*x*_*5*_*, x*_*8*_	The vibration displacements of the front and rear supporting units in the traction direction
*x*_*6*_	The vibration displacement of the shearer body in the traction direction
*λ*	The pitch angle of the shearer
*η*_*z3*_*, η*_*z7*_	The vibration swings of the front and rear haulage unit in plane *xoz*
*β*_*1*_*, β*_*11*_	The vibration swings of the front and rear drums in plane *xoy*
*γ*_*2*_*, γ*_*10*_	The vibration swings of the front and rear ranging arms in plane *xoy*
*α*_*1*_*, α*_*11*_	the lift angles of the front and rear ranging arms for performing lifting operations
*R*_*x1*_*, R*_*y1*_*, R*_*x11*_*, R*_*y11*_	the cutting loads on the front and rear drums in the horizontal and vertical directions
*k*_*x2*_*, k*_*x10*_	the rigidities of the front and rear arms in plane *xoy*
*k*_*qtx*_, *c*_*qtx*_, *k*_*htx*_, *c*_*htx*_	the equivalent rigidities and dampers of the front and rear lift cylinders
*k*_*zx4*_, *c*_*zx4*_, *k*_*zx9*_, *c*_*zx9*_	the equivalent rigidities and resistances of the coupling between the front/rear walking unit and front/rear haulage unit in the traction direction
*k*_*qdx*_, *c*_*qdx*_, *k*_*hdx*_, *c*_*hdx*_	the rigidities and resistances of the contact between the front/rear driving wheel and front/rear pin rail in the traction direction
*k*_*zx5*_, *c*_*zx5*_, *k*_*zx8*_, *c*_*zx8*_	the equivalent rigidities and resistances of the coupling between the rear/front supporting unit and rear/front haulage unit in the traction direction
*k*_*qpx*_, *c*_*qpx*_, *k*_*hpx*_, *c*_*hpx*_	the rigidities and resistances of the contact between the front/rear sliding shoes and the middle trough in the traction direction
*k*_*cms*_, *c*_*cms*_, *k*_*cmx*_, *c*_*cmx*_	the equivalent rigidities and resistances of the upper and lower hydraulic rods on the coalface side
*k*_*cks*_, *c*_*cks*_, *k*_*ckx*_, *c*_*ckx*_	the equivalent rigidities and resistances of the upper and lower hydraulic rods on the goaf side
*e*	the radius of gyration of the gravity center of the ranging arm
*p*	the distance between the gravity centers of the drum and ranging arm
*j*	the width of the traction unit
*b*	the distance between the hinging point of the lift cylinder and ranging arm and the ranging arm rotary pin

Assuming the gaps at the two sides of the coupling between any two shearer components are equal at t = t_0_, the following equation can be obtained by substituting the system kinetic, potential, and dissipated energies as analyzed above into the Lagrange dynamics equation:

3 $${\boldsymbol{M}}\ddot{{\boldsymbol{X}}}+{\boldsymbol{C}}\dot{{\boldsymbol{X}}}+{\boldsymbol{KX}}={\boldsymbol{F}}$$

where matrix ***M*** can be expressed as follows:

$$\begin{array}{rcl}{\boldsymbol{M}} & = & [\begin{array}{ccc}{{\boldsymbol{M}}}_{1} &  & \\  & {{\boldsymbol{M}}}_{2} & \\  &  & {{\boldsymbol{M}}}_{3}\end{array}];\\ {{\boldsymbol{M}}}_{1} & = & [\begin{array}{cccc}{m}_{1}\cdot {p}^{2} & e\cdot {m}_{1}\cdot p\cdot \,\cos \,2{\alpha }_{1} & 0 & -{m}_{1}\cdot p\cdot \,\cos \,{\alpha }_{1}\\ e\cdot {m}_{1}\cdot p\cdot \,\cos \,2{\alpha }_{1} & {e}^{2}\cdot ({m}_{1}+{m}_{2}) & 0 & -e\cdot \,\cos \,{\alpha }_{1}({m}_{1}+{m}_{2})\\ 0 & 0 & {I}_{z3} & 0\\ -{m}_{1}\cdot p\cdot \,\cos \,{\alpha }_{1} & -e\cdot \,\cos \,{\alpha }_{1}({m}_{1}+{m}_{2}) & 0 & {m}_{1}+{m}_{2}+{m}_{3}\end{array}];\\ {{\boldsymbol{M}}}_{2} & = & [\begin{array}{ccc}{m}_{4} & 0 & 0\\ 0 & {m}_{5} & 0\\ 0 & 0 & {m}_{6}\end{array}];\\ {{\boldsymbol{M}}}_{3} & = & [\begin{array}{cccccc}{m}_{7}+{m}_{10}+{m}_{11} & 0 & 0 & 0 & -e\cdot \,\cos \,{\alpha }_{11}({m}_{10}+{m}_{11}) & -{m}_{11}\cdot p\cdot \,\cos \,{\alpha }_{11}\\ 0 & {m}_{8} & 0 & 0 & 0 & 0\\ 0 & 0 & {m}_{9} & 0 & 0 & 0\\ 0 & 0 & 0 & {I}_{z7} & 0 & 0\\ -e\cdot \,\cos \,{\alpha }_{11}({m}_{10}+{m}_{11}) & 0 & 0 & 0 & {e}^{2}\cdot ({m}_{10}+{m}_{11}) & e\cdot {m}_{11}\cdot p\cdot \,\cos \,2{\alpha }_{1}\\ -{m}_{11}\cdot p\cdot \,\cos \,{\alpha }_{11} & 0 & 0 & 0 & e\cdot {m}_{11}\cdot p\cdot \,\cos \,2{\alpha }_{1} & {m}_{11}\cdot {p}^{2}\end{array}];\end{array}$$

where matrix ***C*** can be expressed as follows:

$$\begin{array}{rcl}{\boldsymbol{C}} & = & [\begin{array}{cc}{{\boldsymbol{C}}}_{1} & {{\boldsymbol{C}}}_{3}\\ {{\boldsymbol{C}}}_{4} & {{\boldsymbol{C}}}_{2}\end{array}];\\ {{\boldsymbol{C}}}_{1} & = & \left[\begin{array}{cccccc}0 & 0 & 0 & 0 & 0 & 0\\ 0 & {b}^{2}\cdot {c}_{qtx} & 0 & 0 & 0 & 0\\ 0 & 0 & {C}_{3,3} & {C}_{3,4} & \frac{j}{2}\cdot {c}_{zx4} & \frac{j}{2}\cdot {c}_{zx5}\\ 0 & 0 & {C}_{4,3} & {C}_{4,4} & -{c}_{zx4} & -{c}_{zx5}\\ 0 & 0 & \frac{j}{2}\cdot {c}_{zx4} & -{c}_{zx4} & {c}_{qdx}+{c}_{zx4} & 0\\ 0 & 0 & \frac{j}{2}\cdot {c}_{zx5} & -{c}_{zx5} & 0 & {c}_{qpx}+{c}_{zx5}\end{array}\right];\\ {{\boldsymbol{C}}}_{2} & = & \left[\begin{array}{ccccccc}{C}_{7,7} & {C}_{7,8} & 0 & 0 & {C}_{7,11} & 0 & 0\\ {C}_{8,7} & {C}_{8,8} & -{c}_{zx8} & -{c}_{zx9} & {C}_{8,11} & 0 & 0\\ 0 & -{c}_{zx8} & {c}_{hpx}+{c}_{zx8} & 0 & j\cdot \frac{{c}_{zx8}}{2} & 0 & 0\\ 0 & -{c}_{zx9} & 0 & {c}_{hdx}+{c}_{zx9} & -j\cdot \frac{{c}_{zx9}}{2} & 0 & 0\\ {C}_{11,7} & {C}_{11,8} & j\cdot \frac{{c}_{zx8}}{2} & -j\cdot \frac{{c}_{zx9}}{2} & {C}_{11,11} & 0 & 0\\ 0 & 0 & 0 & 0 & 0 & {b}^{2}\cdot {c}_{htx} & 0\\ 0 & 0 & 0 & 0 & 0 & 0 & 0\end{array}\right];\\ {{\boldsymbol{C}}}_{3} & = & [\begin{array}{ccccccc}0 & 0 & 0 & 0 & 0 & 0 & 0\\ 0 & 0 & 0 & 0 & 0 & 0 & 0\\ {C}_{3,7} & {C}_{3,8} & 0 & 0 & {C}_{3,11} & 0 & 0\\ {C}_{4,7} & {C}_{4,8} & 0 & 0 & {C}_{4,11} & 0 & 0\\ 0 & 0 & 0 & 0 & 0 & 0 & 0\\ 0 & 0 & 0 & 0 & 0 & 0 & 0\end{array}];\\ {{\boldsymbol{C}}}_{4} & = & [\begin{array}{cccccc}0 & 0 & {C}_{7,3} & {C}_{7,4} & 0 & 0\\ 0 & 0 & {C}_{8,3} & {C}_{8,4} & 0 & 0\\ 0 & 0 & 0 & 0 & 0 & 0\\ 0 & 0 & 0 & 0 & 0 & 0\\ 0 & 0 & {C}_{11,3} & {C}_{11,4} & 0 & 0\\ 0 & 0 & 0 & 0 & 0 & 0\\ 0 & 0 & 0 & 0 & 0 & 0\end{array}];\end{array}$$

$$\begin{array}{ccc}{C}_{3,3} & = & \frac{{j}^{2}}{2}\cdot ({c}_{cks}+{c}_{ckx}+{c}_{cms}+{c}_{cmx}+\frac{{c}_{zx4}+{c}_{zx5}}{2});\\ {C}_{3,4} & = & -({c}_{ckx}-{c}_{cks}-{c}_{cms}+{c}_{cmx}+\frac{{c}_{zx4}+{c}_{zx5}}{2})\end{array}$$

$$\begin{array}{ccc}{C}_{3,7} & = & j\cdot ({c}_{ckx}-{c}_{cks}-{c}_{cms}+{c}_{cmx});\\ {C}_{3,8} & = & j\cdot ({c}_{ckx}-{c}_{cks}-{c}_{cms}+{c}_{cmx});\\ {C}_{3,11} & = & \frac{{j}^{2}}{2}\cdot ({c}_{cks}+{c}_{ckx}+{c}_{cms}+{c}_{cmx})\end{array}$$

$$\begin{array}{ccc}{C}_{4,3} & = & -j\cdot ({c}_{ckx}-{c}_{cks}-{c}_{cms}+{c}_{cmx}+\frac{{c}_{zx4}+{c}_{zx5}}{2});\\ {C}_{4,4} & = & 2\cdot ({c}_{cks}+{c}_{ckx}+{c}_{cms}+{c}_{cmx})+{c}_{zx4}+{c}_{zx5};\end{array}$$

$$\begin{array}{ccc}{C}_{4,7} & = & -2\cdot ({c}_{cks}+{c}_{ckx}+{c}_{cms}+{c}_{cmx});\\ {C}_{4,8} & = & -2\cdot ({c}_{cks}+{c}_{ckx}+{c}_{cms}+{c}_{cmx});\\ {C}_{4,11} & = & -j\cdot ({c}_{ckx}-{c}_{cks}-{c}_{cms}+{c}_{cmx});\end{array}$$

$$\begin{array}{ccc}{C}_{7,3} & = & j\cdot ({c}_{ckx}-{c}_{cks}-{c}_{cms}+{c}_{cmx});\\ {C}_{7,4} & = & -2\cdot ({c}_{cks}+{c}_{ckx}+{c}_{cms}+{c}_{cmx});\\ {C}_{7,7} & = & 2\cdot ({c}_{cks}+{c}_{ckx}+{c}_{cms}+{c}_{cmx});\end{array}$$

$$\begin{array}{ccc}{C}_{7,8} & = & 2\cdot ({c}_{cks}+{c}_{ckx}+{c}_{cms}+{c}_{cmx});\\ {C}_{7,11} & = & j\cdot ({c}_{ckx}-{c}_{cks}-{c}_{cms}+{c}_{cmx});\\ {C}_{8,3} & = & j\cdot ({c}_{ckx}-{c}_{cks}-{c}_{cms}+{c}_{cmx});\end{array}$$

$$\begin{array}{ccc}{C}_{8,4} & = & -2\cdot ({c}_{cks}+{c}_{ckx}+{c}_{cms}+{c}_{cmx});\\ {C}_{8,7} & = & 2\cdot ({c}_{cks}+{c}_{ckx}+{c}_{cms}+{c}_{cmx});\end{array}$$

$$\begin{array}{ccc}{C}_{8,8} & = & 2\cdot ({c}_{cks}+{c}_{ckx}+{c}_{cms}+{c}_{cmx})+{c}_{zx8}+{c}_{zx9};\\ {C}_{8,11} & = & j\cdot ({c}_{ckx}-{c}_{cks}-{c}_{cms}+{c}_{cmx}+\frac{-{c}_{zx8}+{c}_{zx9}}{2});\end{array}$$

$$\begin{array}{ccc}{C}_{11,3} & = & \frac{{j}^{2}}{2}\cdot ({c}_{cks}+{c}_{ckx}+{c}_{cms}+{c}_{cmx});\\ {C}_{11,4} & = & -j\cdot ({c}_{ckx}-{c}_{cks}-{c}_{cms}+{c}_{cmx});\\ {C}_{11,7} & = & j\cdot ({c}_{ckx}-{c}_{cks}-{c}_{cms}+{c}_{cmx});\end{array}$$

$$\begin{array}{ccc}{C}_{11,8} & = & j\cdot ({c}_{ckx}-{c}_{cks}-{c}_{cms}+{c}_{cmx}+\frac{-{c}_{zx8}+{c}_{zx9}}{2});\\ {C}_{11,11} & = & \frac{{j}^{2}}{2}\cdot ({c}_{cks}+{c}_{ckx}+{c}_{cms}+{c}_{cmx}+\frac{{c}_{zx8}+{c}_{zx9}}{2});\end{array}$$

where matrix ***K*** can be expressed as follows:

$${\boldsymbol{K}}=[\begin{array}{cc}{{\boldsymbol{K}}}_{1} & {{\boldsymbol{K}}}_{3}\\ {{\boldsymbol{K}}}_{4} & {{\boldsymbol{K}}}_{2}\end{array}]$$

$$\begin{array}{c}{{\boldsymbol{K}}}_{1}=\left[\begin{array}{cccccc}{p}^{2}\cdot {k}_{x2} & 0 & 0 & 0 & 0 & 0\\ 0 & {b}^{2}\cdot {k}_{qtx} & 0 & 0 & 0 & 0\\ 0 & 0 & {K}_{3,3} & {K}_{3,4} & \frac{j}{2}\cdot {k}_{zx4} & \frac{j}{2}\cdot {k}_{zx5}\\ 0 & 0 & {K}_{4,3} & {K}_{4,4} & -{k}_{zx4} & -{k}_{zx5}\\ 0 & 0 & \frac{j}{2}\cdot {k}_{zx4} & -{k}_{zx4} & {k}_{qdx}+{k}_{zx4} & 0\\ 0 & 0 & \frac{j}{2}\cdot {k}_{zx5} & -{k}_{zx5} & 0 & {k}_{qpx}+{k}_{zx5}\end{array}\right];\\ {{\boldsymbol{K}}}_{2}=\left[\begin{array}{ccccccc}{K}_{7,7} & {K}_{7,8} & 0 & 0 & {K}_{7,11} & 0 & 0\\ {K}_{8,7} & {K}_{8,8} & -{k}_{zx8} & -{k}_{zx9} & {K}_{8,11} & 0 & 0\\ 0 & -{k}_{zx8} & {k}_{hpx}+{k}_{zx8} & 0 & j\cdot \frac{{k}_{zx8}}{2} & 0 & 0\\ 0 & -{k}_{zx9} & 0 & {k}_{hdx}+{k}_{zx9} & -j\cdot \frac{{k}_{zx9}}{2} & 0 & 0\\ {K}_{11,7} & {K}_{11,8} & j\cdot \frac{{k}_{zx8}}{2} & -j\cdot \frac{{k}_{zx9}}{2} & {K}_{11,11} & 0 & 0\\ 0 & 0 & 0 & 0 & 0 & {b}^{2}\cdot {k}_{htx} & 0\\ 0 & 0 & 0 & 0 & 0 & 0 & {p}^{2}\cdot {k}_{x10}\end{array}\right];\end{array}$$

$$\begin{array}{ccc}{{\boldsymbol{K}}}_{3} & = & [\begin{array}{ccccccc}0 & 0 & 0 & 0 & 0 & 0 & 0\\ 0 & 0 & 0 & 0 & 0 & 0 & 0\\ {K}_{3,7} & {K}_{3,8} & 0 & 0 & {K}_{3,11} & 0 & 0\\ {K}_{4,7} & {K}_{4,8} & 0 & 0 & {K}_{4,11} & 0 & 0\\ 0 & 0 & 0 & 0 & 0 & 0 & 0\\ 0 & 0 & 0 & 0 & 0 & 0 & 0\end{array}];\\ {{\boldsymbol{K}}}_{4} & = & [\begin{array}{cccccc}0 & 0 & {K}_{7,3} & {K}_{7,4} & 0 & 0\\ 0 & 0 & {K}_{8,3} & {K}_{8,4} & 0 & 0\\ 0 & 0 & 0 & 0 & 0 & 0\\ 0 & 0 & 0 & 0 & 0 & 0\\ 0 & 0 & {K}_{11,3} & {K}_{11,4} & 0 & 0\\ 0 & 0 & 0 & 0 & 0 & 0\\ 0 & 0 & 0 & 0 & 0 & 0\end{array}];\end{array}$$

$$\begin{array}{ccc}{K}_{3,3} & = & \frac{{j}^{2}}{2}\cdot ({k}_{cks}+{k}_{ckx}+{k}_{cms}+{k}_{cmx}+\frac{{k}_{zx4}+{k}_{zx5}}{2});\\ {K}_{3,4} & = & j\cdot ({k}_{cks}-{k}_{ckx}+{k}_{cms}-{k}_{cmx}-\frac{{k}_{zx4}+{k}_{zx5}}{2});\end{array}$$

$$\begin{array}{ccc}{K}_{3,7} & = & j\cdot ({k}_{ckx}-{k}_{cks}-{k}_{cms}+{k}_{cmx});{K}_{3,8}=j\cdot ({k}_{ckx}-{k}_{cks}-{k}_{cms}+{k}_{cmx});\\ {K}_{3,11} & = & \frac{{j}^{2}}{2}\cdot ({k}_{cks}+{k}_{ckx}+{k}_{cms}+{k}_{cmx});\end{array}$$

$$\begin{array}{ccc}{K}_{4,3} & = & j\cdot ({k}_{cks}-{k}_{ckx}+{k}_{cms}-{k}_{cmx}-\frac{{k}_{zx4}+{k}_{zx5}}{2});\\ {K}_{4,4} & = & 2\cdot ({k}_{cks}+{k}_{ckx}+{k}_{cms}+{k}_{cmx})+{k}_{zx4}+{k}_{zx5};\end{array}$$

$$\begin{array}{ccc}{K}_{4,7} & = & -2\cdot ({k}_{cks}+{k}_{ckx}+{k}_{cms}+{k}_{cmx});{K}_{4,8}=-2\cdot ({k}_{cks}+{k}_{ckx}+{k}_{cms}+{k}_{cmx});\\ {K}_{4,11} & = & j\cdot ({k}_{cks}-{k}_{ckx}+{k}_{cms}-{k}_{cmx})\end{array}$$

$$\begin{array}{ccc}{K}_{7,3} & = & j\cdot ({k}_{ckx}-{k}_{cks}-{k}_{cms}+{k}_{cmx});{K}_{7,4}=-2\cdot ({k}_{cks}+{k}_{ckx}+{k}_{cms}+{k}_{cmx});\\ {K}_{7,7} & = & 2\cdot ({k}_{cks}+{k}_{ckx}+{k}_{cms}+{k}_{cmx});\end{array}$$

$$\begin{array}{ccc}{K}_{7,8} & = & 2\cdot ({k}_{cks}+{k}_{ckx}+{k}_{cms}+{k}_{cmx});{K}_{7,11}=j\cdot ({k}_{ckx}-{k}_{cks}-{k}_{cms}+{k}_{cmx});\\ {K}_{8,3} & = & j\cdot ({k}_{ckx}-{k}_{cks}-{k}_{cms}+{k}_{cmx})\end{array}$$

$${K}_{8,4}=-\,2\cdot ({k}_{cks}+{k}_{ckx}+{k}_{cms}+{k}_{cmx});{K}_{8,7}=\,2\cdot ({k}_{cks}+{k}_{ckx}+{k}_{cms}+{k}_{cmx});$$

$$\begin{array}{ccc}{K}_{8,8} & = & 2\cdot ({k}_{cks}+{k}_{ckx}+{k}_{cms}+{k}_{cmx})+{k}_{zx8}+{k}_{zx9};\\ {K}_{8,11} & = & j\cdot ({k}_{ckx}-{k}_{cks}-{k}_{cms}+{k}_{cmx}+\frac{-{k}_{zx8}+{k}_{zx9}}{2});\end{array}$$

$$\begin{array}{ccc}{K}_{11,3} & = & \frac{{j}^{2}}{2}\cdot ({k}_{cks}+{k}_{ckx}+{k}_{cms}+{k}_{cmx});{K}_{11,4}=j\cdot ({k}_{cks}-{k}_{ckx}+{k}_{cms}-{k}_{cmx});\\ {K}_{11,7} & = & j\cdot ({k}_{ckx}-{k}_{cks}-{k}_{cms}+{k}_{cmx});\end{array}$$

$$\begin{array}{ccc}{K}_{11,8} & = & j\cdot ({k}_{ckx}-{k}_{cks}-{k}_{cms}+{k}_{cmx}+\frac{-{k}_{zx8}+{k}_{zx9}}{2});\\ {K}_{11,11} & = & \frac{{j}^{2}}{2}\cdot ({k}_{cks}+{k}_{ckx}+{k}_{cms}+{k}_{cmx}+\frac{{k}_{zx8}+{k}_{zx9}}{2}).\end{array}$$

here matrix ***X*** can be expressed as follows:

$${\boldsymbol{X}}={[{\beta }_{1},{\gamma }_{2},{\eta }_{z3},{x}_{3},{x}_{4},{x}_{5},{x}_{6},{x}_{7},{x}_{8},{x}_{9},{\eta }_{z7},{\gamma }_{10},{\beta }_{11}]}^{T}$$

and matrix ***F*** can be expressed as follows:

$${\boldsymbol{F}}={\left[-{R}_{y1}p\cos ({\alpha }_{1}+\lambda )-{R}_{x1}p\sin ({\alpha }_{1}+\lambda ),0,0,0,\frac{1}{2}{d}_{xz}{k}_{qdx},0,0,0,0,\frac{1}{2}{d}_{xz}{k}_{hdx},0,0,{R}_{y11}p\cos ({\alpha }_{11}+\lambda )+{R}_{x11}p\sin ({\alpha }_{11}+\lambda )\right]}^{T}$$

### Numerical simulation of the rigidity of critical shearer components

#### Tangential rigidity of coupling between sliding shoe and middle trough

**Figure 3:**
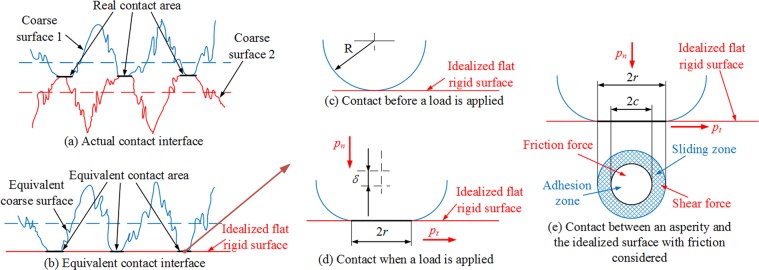
Illustration of the contact between the sliding shoe (assumed a coarse surface with asperities) and the middle trough (assumed an idealized flat surface).

On nominally flat, rigid surfaces obtained through mechanical machining, no matter the machining accuracy, there are many micro-asperities of different sizes and shapes. When the contact between two flat surfaces is subjected to a mechanical impact, these asperities deform: elastic, elastoplastic, and plastic. Only elastic deformation was considered here. The contact between the sliding shoe (supporting unit) and the middle trough (scraper conveyor) was characterized using the Greenwood-Williamson (GW) model^20^ and Chang-Etsion-Bogy (CEB) model^21^, as illustrated in Fig. 3(a). Because the contact between the two components consists of the contacts between the asperities on the two touching surfaces, the touching surface of the sliding shoe was assumed a coarse surface with asperities, whereas the middle trough was assumed an idealized flat surface, as shown in Fig. 3(b). In the contact between the asperities and the idealized surface, the asperities were assumed approximately spherical. Figure 3(c,d) illustrate the contact between the spherical asperities and the idealized surface before and after a load is applied. In friction, the shear stress at the contact interface approximates infinite, whereas the normal stress is low. Only with a tangential load larger than the maximum static friction can sliding occur at the interface. Thus, the contact area between a single asperity on the sliding shoe and the idealized middle trough surface can be divided into a sticking zone and a sliding zone, as shown in Fig. 3(e).

The authors used both the Hertz contact theory and the model of Li *et al*.^22^ to describe the tangential rigidity and friction of contact interfaces. The tangential rigidity of the contact between the front/rear sliding shoes and the scraper conveyor middle trough with only elastic deformation at the contact interface considered can then be expressed as follows:

4 $${k}_{qpx}={\left(\frac{3}{4\pi }\right)}^{\frac{1}{3}}\cdot \frac{4D}{1-D}\cdot \frac{\frac{{G}_{s}\cdot {P}_{tqp}}{\mu {P}_{nqp}}}{1-{\left(1-\frac{{P}_{tqp}}{\mu {P}_{nqp}}\right)}^{\frac{2}{3}}}\cdot \left(\frac{{P}_{nqp}}{{E}_{pz}\cdot {A}_{pz}}\right)\cdot {\psi }^{1-0.5D}{G}^{\frac{1-D}{3}}{a}_{\max }^{0.5D}({a}_{pz}^{\frac{1-D}{3}}-{a}_{\mu c}^{\frac{1-D}{3}})$$

5 $${k}_{hpx}={\left(\frac{3}{4\pi }\right)}^{\frac{1}{3}}\cdot \frac{4D}{1-D}\cdot \frac{\frac{{G}_{s}\cdot {P}_{thp}}{\mu {P}_{nqp}}}{1-{\left(1-\frac{{P}_{thp}}{\mu {P}_{nhp}}\right)}^{\frac{2}{3}}}\cdot \left(\frac{{P}_{nhp}}{{E}_{pz}\cdot {A}_{pz}}\right)\cdot {\psi }^{1-0.5D}{G}^{\frac{1-D}{3}}{a}_{\max }^{0.5D}({a}_{pz}^{\frac{1-D}{3}}-{a}_{\mu c}^{\frac{1-D}{3}})$$

6 $${E}_{pz}={\left(\frac{1-{\upsilon }_{p}^{2}}{{E}_{p}}+\frac{1-{\upsilon }_{z}^{2}}{{E}_{z}}\right)}^{-1}$$

where *D* is the fractal dimension; *G*_*s*_ is the equivalent shear modulus of a single micro-asperities; *μ* is the coefficient of friction; *G* is the fractal roughness parameter^23^; *ψ* is the spreading factor of micro-asperities contact area distribution, with its value (*ψ* > 1) related to fractal dimension D^24^; *E*_*pz*_ is the equivalent modulus of elasticity; *E*_*p*_ and *E*_*z*_ are the moduli of elasticity of the sliding shoe and middle trough, respectively; *υ*_*p*_ and *υ*_*z*_ are the Poisson ratios of the sliding shoe and middle trough, respectively; *P*_*tqp*_ and *P*_*thp*_ are the tangential loads on the contacts between the front/rear sliding shoes and the middle trough in the traction direction, respectively; *P*_*nqp*_ and *P*_*nhp*_ are the normal loads on the contacts between the front/rear sliding shoes and the middle trough in the traction direction, respectively; *A*_*pz*_ is the real contact area between the sliding shoe and middle trough; *a*_*pz*_ is the real contact area between the micro-asperities on the sliding shoe and the middle trough; *a*_*max*_ is the maximum contact area between the individual micro-asperities on the sliding shoe and the middle trough; and *a*_*μc*_ is the contact area between the micro-asperities on the sliding shoe and the middle trough at the critical point of the transition from elastic to plastic deformation.

#### Normal rigidity of the gapped contact between driving wheel and pin rail

**Figure 4:**
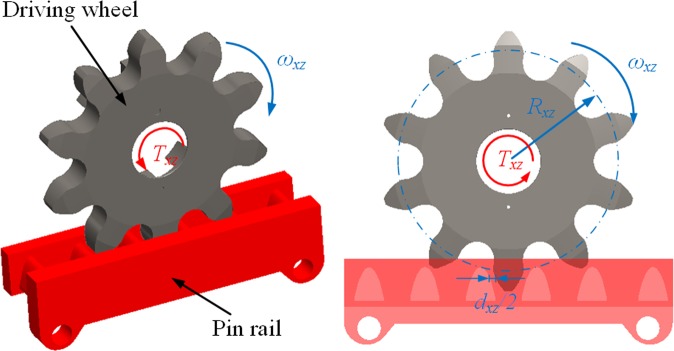
Model for describing the contact between the driving wheel and pin rail.

The contact between the driving wheel and scraper conveyor pin rail is a gapped one. Figure 4 illustrates the gaps (designated as *d*_*xz*_) between the driving wheel and the side walls of the pin rail at t = t_0_.

The wheel-pin contact can be considered the contact between two meshing gears. Thus, with the gap at the contact considered, the normal contact between the driving wheel and pin rail can be expressed as follows^25^:

7 $${K}_{nx}=\{\begin{array}{cc}\frac{{T}_{xz}}{{R}_{xz}{\delta }_{nx}} & |[{x}_{i}-{e}_{i}(t)]-{\omega }_{xz}{R}_{xz}| > \frac{{d}_{xz}}{2}\\ 0 & |[{x}_{i}-{e}_{i}(t)]-{\omega }_{xz}{R}_{xz}|\le \frac{{d}_{xz}}{2}\end{array}(i=4,9)$$

where *T*_*xz*_ is the torsional torque of the driving wheel; *R*_*xz*_ is the radius of the reference circle of the driving wheel; *δ*_*nx*_ is the normal deformation at the wheel-pin contact; *ω*_*xz*_ is the angular velocity of the driving wheel; *x*_*i*_ is the vibration displacement of the front/rear driving wheels; and *e*_i_(*t*) is the gear frequency error function.

The normal deformation at the contact between the driving wheel and pin rail, *δ*_*nx*_, consists of the plastic bending of the driving wheel, the plastic deformation of the wheel hub, the change in the tooth contact position caused by the plastic deformation of the axis and supporting structure, and the local elastic deformation at the contact between meshing teeth. The present study only considered the effect of the elastic deformation on the rigidity of the contact between the driving wheel and pin rail. Thus, the normal deformation, *δ*_*nx*_, can be expressed as follows:

8 $${\delta }_{nx}=\left\{\begin{array}{ll}{\omega }_{xz}{R}_{xz}-\frac{{d}_{xz}}{2}-\left[{x}_{i}+{e}_{i}(t)\right] & \left[{x}_{i}-{e}_{i}(t)\right]-{\omega }_{xz}{R}_{xz}\le -\frac{{d}_{xz}}{2}\\ 0 & -\frac{{d}_{xz}}{2} < \left[{x}_{i}-{e}_{i}(t)\right]-{\omega }_{xz}{R}_{xz}\le \frac{{d}_{xz}}{2}\\ \left[{x}_{i}+{e}_{i}(t)\right]-\frac{{d}_{xz}}{2}-{\omega }_{xz}{R}_{xz} & [{x}_{i}-{e}_{i}(t)]-{\omega }_{xz}{R}_{xz} > \frac{{d}_{xz}}{2}\end{array}\right.(i=4,9)$$

Thus, the rigidity of the contact between the front/rear driving wheels and the scraper conveyor pin rail can be expressed as follows:

9 $${k}_{qdx}=\{\begin{array}{cc}\frac{{T}_{xz}}{{R}_{xz}\left\{{\omega }_{xz}{R}_{xz}-\frac{{d}_{xz}}{2}-[{x}_{4}+{e}_{4}(t)]\right\}} & [{x}_{4}-{e}_{4}(t)]-{\omega }_{xz}{R}_{xz}\le -\frac{{d}_{xz}}{2}\\ 0 & -\frac{{d}_{xz}}{2} < [{x}_{4}-{e}_{4}(t)]-{\omega }_{xz}{R}_{xz}\le \frac{{d}_{xz}}{2}\\ \frac{{T}_{xz}}{{R}_{xz}\left\{[{x}_{4}+{e}_{4}(t)]-\frac{{d}_{xz}}{2}-{\omega }_{xz}{R}_{xz}\right\}} & [{x}_{4}-{e}_{4}(t)]-{\omega }_{xz}{R}_{xz} > \frac{{d}_{xz}}{2}\end{array}$$

10 $${k}_{hdx}=\{\begin{array}{cc}\frac{{T}_{xz}}{{R}_{xz}\left\{{\omega }_{xz}{R}_{xz}-\frac{{d}_{xz}}{2}-[{x}_{9}+{e}_{9}(t)]\right\}} & [{x}_{9}-{e}_{9}(t)]-{\omega }_{xz}{R}_{xz}\le -\frac{{d}_{xz}}{2}\\ 0 & -\frac{{d}_{xz}}{2} < [{x}_{9}-{e}_{9}(t)]-{\omega }_{xz}{R}_{xz}\le \frac{{d}_{xz}}{2}\\ \frac{{T}_{xz}}{{R}_{xz}\left\{[{x}_{9}+{e}_{9}(t)]-\frac{{d}_{xz}}{2}-{\omega }_{xz}{R}_{xz}\right\}} & [{x}_{9}-{e}_{9}(t)]-{\omega }_{xz}{R}_{xz} > \frac{{d}_{xz}}{2}\end{array}$$

#### Rigidity of lift cylinder

**Figure 5:**
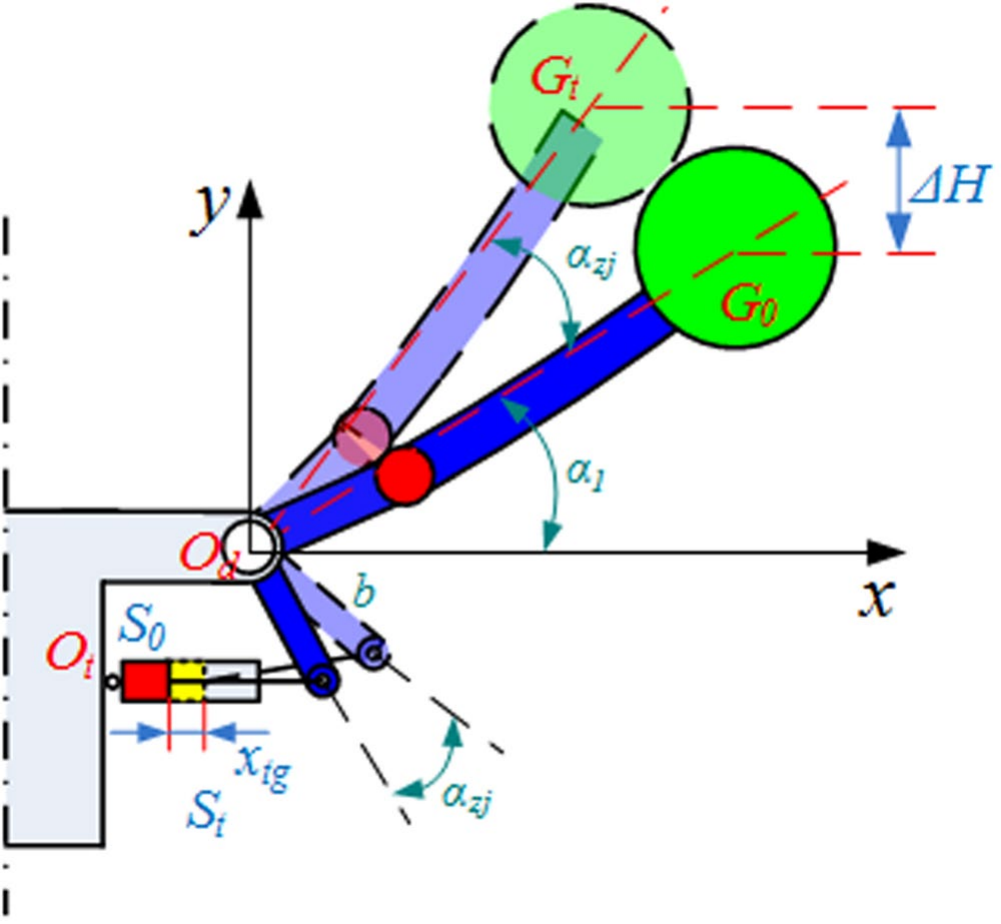
Illustration of drum height adjustment mechanism.

A change in the travel of the lift cylinder results in a change in the shearer lift angle, thereby altering the height of the drum to cut into the coalface at different heights. Thus, the change in the lift cylinder travel affects its equivalent supporting rigidity. Figure 5 illustrates the height adjustment mechanism of the front cutting unit, where a change in the cylinder travel results in a rotation of the arm around point *O*_*d*_ and a change of the drum position from *G*_*0*_ at the initial moment to *G*_*t*_.

Thus, the change in the lift cylinder travel, *x*_*tg*_, can be expressed as follows:

11 $${x}_{tg}={S}_{t}-{S}_{0}=b\cdot \left(\frac{\Delta H}{b}+\,\sin \,{\alpha }_{1}\right)\cos \,\frac{{\alpha }_{1}}{2}-\,2(e+p)\sin \,\frac{{\alpha }_{1}}{2}\,\cos \,\frac{\arcsin \left(\frac{\Delta H}{b}\,\sin \,{\alpha }_{1}\right)}{2}$$

12 $$\Delta H=b\cdot [\sin ({\alpha }_{1}+{\alpha }_{zj})-\,\sin \,{\alpha }_{1}]$$

where *x*_*tg*_ is the increment in the cylinder travel; *S*_1_ is the initial travel of the lift cylinder of the front cutting unit; *S*_*t*_ is the travel of the lift cylinder when the drum is lifted to position *G*_*t*_; *ΔH* is the increment in the drum cutting height; and *α*_*qz*_ is the increment in the lift angle of the front cutting unit.

Thus, the equivalent rigidity of the lift cylinder of the front/rear cutting units can be expressed as follows:

13 $${k}_{qtx}=\frac{4{\beta }_{e}{B}_{m}}{{V}_{m}({S}_{1}+{x}_{tg})}=\frac{4{\beta }_{e}{B}_{m}}{{V}_{m}\left({S}_{1}+(e+p)\sin ({\alpha }_{1}+{\alpha }_{qz})\cdot \,\cos \,\frac{{\alpha }_{1}}{2}-\,2(e+p)\sin \,\frac{{\alpha }_{1}}{2}\cdot \,\cos \,\frac{\arcsin (\sin ({\alpha }_{1}+{\alpha }_{qz})\cdot \,\sin \,{\alpha }_{1}-{\sin }^{2}{\alpha }_{1})}{2}\right)}$$

14 $${k}_{htx}=\frac{4{\beta }_{e}{B}_{m}}{{V}_{m}\left({S}_{1}+(e+p)\sin ({\alpha }_{11}+{\alpha }_{hz})\cdot \,\cos \,\frac{{\alpha }_{11}}{2}-\,2(e+p)\sin \,\frac{{\alpha }_{11}}{2}\cdot \,\cos \,\frac{\arcsin (\sin ({\alpha }_{11}+{\alpha }_{hz})\cdot \,\sin \,{\alpha }_{11}-{\sin }^{2}{\alpha }_{11})}{2}\right)}$$

where *β*_*e*_ is the effective modulus of volume elasticity of the lift cylinder; *B*_*m*_ is the average of the effective areas of the two cavities of the lift cylinder; *V*_*m*_ is the average of the equivalent total volumes of the two cavities of the lift cylinder; and *α*_*hz*_ is the increment in the lift angle of the rear cutting unit.

#### Rigidity of ranging arm

With reference to the shearer arm rigidity model proposed by Xie *et al*.^26^, the equivalent rigidity of the shearer ranging arm considered in the present study can be expressed as follows:

15 $${k}_{x2}={k}_{x10}=\frac{3{E}_{e}{I}_{e}}{{p}^{3}}$$

#### Rigidity of the coupling between the shearer body and haulage unit

The shearer body and the traction unit were coupled through four hydraulic rods. Thus, the rigidity of the coupling can be indicated by the rigidity of the four rods. The hydraulic rods are required to be pre-tensioned during the shearer assembly process. The rods may not experience tension and compression—with its rigidity equal to zero—under certain field operating conditions due to the vibration-induced swing/displacement of the shearer body and traction unit. The rigidity of the four hydraulic rods under these conditions was characterized. Table 4 shows the relevant parameters of the hydraulic rods.

**Table 4:** Relevant parameters of hydraulic rod.

Designation of rod	Position of rod	Screw specification	maximum rod travel /mm	Rod travel for pre-tension/mm
*l*_*ckx*_	Lower goaf	M56 × 4	3460	6
*l*_*cks*_	Upper goaf	M56 × 4	4370	7
*l*_*cmx*_	Lower coalface	M56 × 4	3460	6
*l*_*cms*_	Upper coalface	M56 × 4	6080	10

Thus, the rigidity of the hydraulic rods can be expressed as:

16 $${k}_{cms}=\{\begin{array}{cc}\frac{3{E}_{e}{I}_{e}}{{l}_{cms}^{3}} & {x}_{7}-{x}_{3}-\frac{j}{2}\cdot ({\eta }_{z3}+{\eta }_{z7})\ne 10\\ 0 & {x}_{7}-{x}_{3}-\frac{j}{2}\cdot ({\eta }_{z3}+{\eta }_{z7})=10\end{array}$$

17 $${k}_{cmx}=\{\begin{array}{cc}\frac{3{E}_{e}{I}_{e}}{{l}_{cmx}^{3}} & {x}_{7}-{x}_{3}-\frac{j}{2}\cdot ({\eta }_{z3}+{\eta }_{z7})\ne 6\\ 0 & {x}_{7}-{x}_{3}-\frac{j}{2}\cdot ({\eta }_{z3}+{\eta }_{z7})=6\end{array}$$

18 $${k}_{cks}=\{\begin{array}{cc}\frac{3{E}_{e}{I}_{e}}{{l}_{cks}^{3}} & {x}_{7}-{x}_{3}-\frac{j}{2}\cdot ({\eta }_{z3}+{\eta }_{z7})\ne 7\\ 0 & {x}_{7}-{x}_{3}-\frac{j}{2}\cdot ({\eta }_{z3}+{\eta }_{z7})=7\end{array}$$

19 $${k}_{ckx}=\{\begin{array}{cc}\frac{3{E}_{e}{I}_{e}}{{l}_{ckx}^{3}} & {x}_{7}-{x}_{3}-\frac{j}{2}\cdot ({\eta }_{z3}+{\eta }_{z7})\ne 6\\ 0 & {x}_{7}-{x}_{3}-\frac{j}{2}\cdot ({\eta }_{z3}+{\eta }_{z7})=6\end{array}$$

where *I*_*e*_ is the cross-sectional moment of inertia of the hydraulic rod.

## Simulation Results and Analysis

The shearer traction-swing coupling dynamics equations were resolved to obtain the dynamic responses of the critical shear components (drums, ranging arms, walking units, supporting units, and body).

### Vibration displacement/swing

With the parameters deemed to optimize economic performance of shearers in the field, the dynamical equations were solved using the following shearer settings: traction speed: 3 m/min; drum cutting depth: 600 mm; drum rotational speed: 32 rpm; pitch angle: 0; lift angle of the front ranging arm: 27°; lift angle of rear ranging arm: −15°; coalface hardness coefficient: *f* = 3.

**Figure 6:**
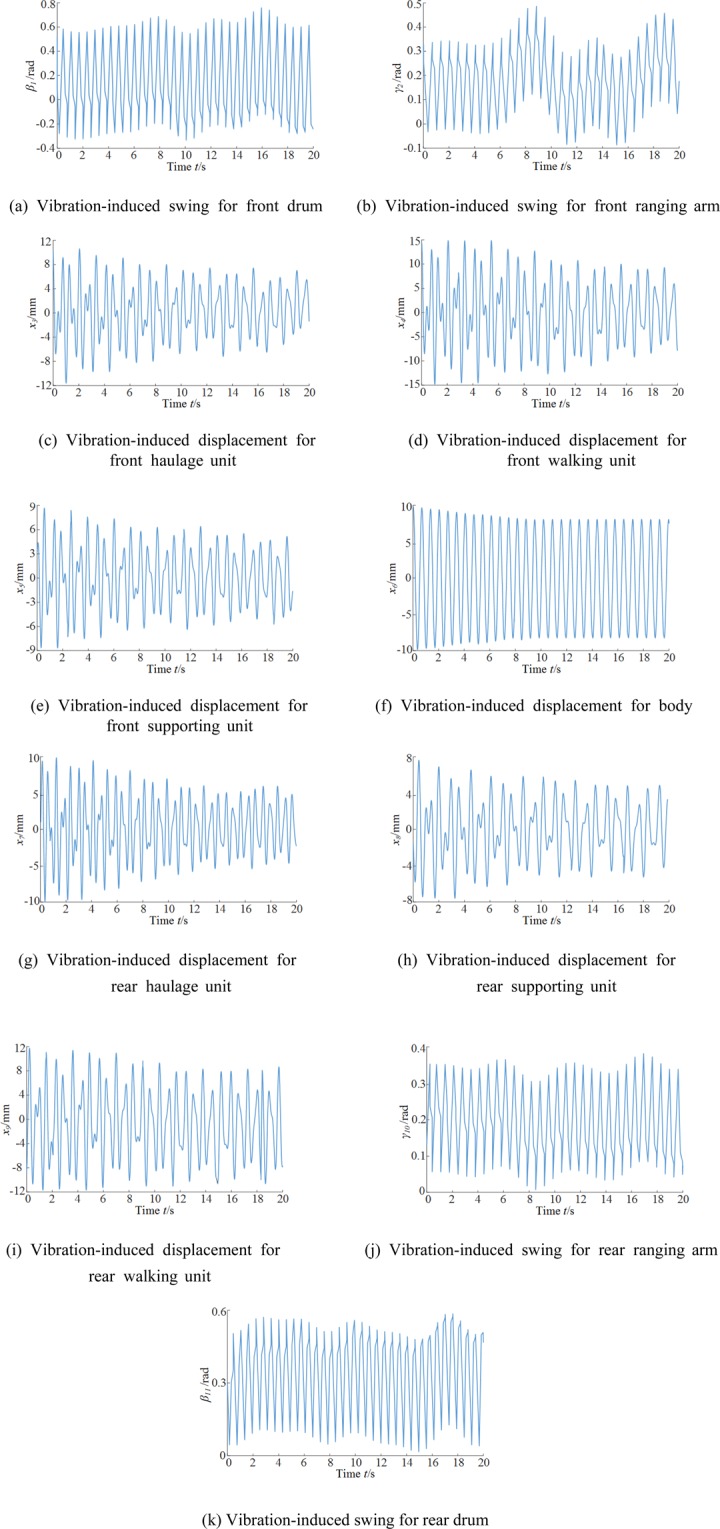
Time curves for the vibration-induced displacements/swings of critical shearer components. (**a**) Vibration-induced swing for front drum (**b**) Vibration-induced swing for front ranging arm. (**c**) Vibration-induced displacement for (**d**) Vibration-induced displacement for front haulage unit front walking unit (**e**) Vibration-induced displacement for (**f**) Vibration-induced displacement for body front supporting unit (**g**) Vibration-induced displacement for (**h**) Vibration-induced displacement for rear haulage unit rear supporting unit (**i**) Vibration-induced displacement for (**j**) Vibration-induced swing for rear ranging arm rear walking unit (**k**) Vibration-induced swing for rear drum.

The vibration-induced displacements/swings of the critical components were simulated for a period of 20 s, with the results presented in Fig. 6. Table 5 shows the masses of the components.

**Table 5:** Related parameters of shearer components.

Mass/kg						Length/m			
m_1_, m_11_	m_2_, m_10_	m_3_, m_7_	m_4_, m_9_	m_5_, m_8_	m_6_	e	p	j	b
5 × 10^3^	8.5 × 10^3^	9.6 × 10^3^	3.2 × 10^3^	2.5 × 10^3^	19.3 × 10^3^	0.93	1.86	1.5	0.5

The vibration-induced displacements/swings of the front drum, ranging arm, haulage unit, walking unit, and supporting unit were larger than those of their rear counterparts, because the front drum was subject to a larger load than the rear. The vibration swings of the front and rear cutting units were large, because the front and rear drums served as the input terminals for the external excitation on the whole shearer system. The front and rear drum were simulated to swing between −0.4 and 0.8 rad, and 0 and 0.6 rad; the front and rear ranging arms were simulated to swing from −0.1–0.5 rad and from 0–0.4 rad.

Due to the different lift angles of the front and rear cutting units, the front and rear lift cylinders had different equivalent rigidities. This led to a phase lap between the time-domain responses of the front and rear components of the shearer. As the vibration propagated from the drums to other components in the shear system, the phase lap between shearer components gradually decreased. The response time curve of the heavy shearer body showed stable variations.

The vibration displacements of the walking units, supporting units, and body varied symmetrically around the horizontal axis (displacement = 0 mm). The model of the contact between the driving wheel and pin rail assumed the gaps between the wheel and pin rail walls equal and the rigidities between the body and the supporting and walking units large. The displacements of the front and rear walking units varied by ±12 and ±10 mm, respectively; those of the supporting units varied by ±6 mm; those of the front and rear haulage units varied by ±8 and ±7 mm, respectively; and the body exhibited displacements of ±8 mm.

### Analysis of vibration acceleration behavior

The above analysis shows the front components of the shearer were subject to larger vibrations and the vibration displacements of the front haulage, supporting, and walking units varied in similar patterns. Considering that the walking and cutting units are critical to the walking and coal cutting operations of the shearer, and the gapped coupling between these two shearer components directly impacts the shear vibration acceleration, the vibration accelerations of the front drum, ranging arm, and walking unit were analyzed as follows.

**Figure 7:**
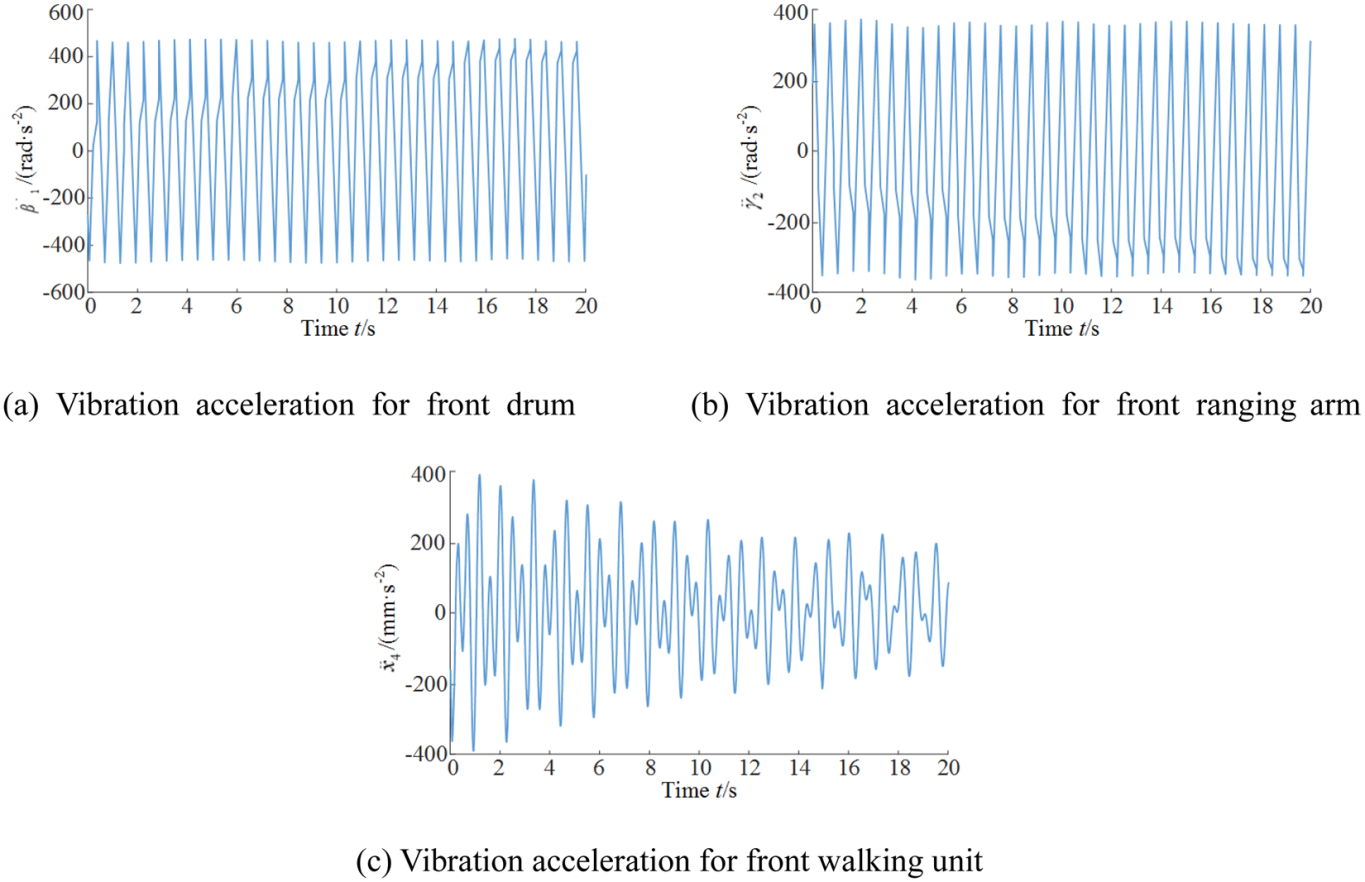
Vibration acceleration time curves for critical shearer components. (**a**) Vibration acceleration for front drum (**b**) Vibration acceleration for front ranging arm. (**c**) Vibration acceleration for front walking unit.

As shown in Fig. 7(a,b), the drums served as the input terminals for the load on the entire dynamical system and are thus subject to the largest load. The vibration acceleration of the front drum varied ±450 rad/s^2^. The ranging arms that were directly coupled with the drum were subject to inertia; their acceleration was influenced by the drums. The vibration acceleration of the front arm exhibited a similar variation pattern with the drums at ±380 rad/s^2^.

From the description of the contact between the driving wheel and pin rail above, the wheel-pin contact was divided into three conditions, namely no contact, contact, and impact. As shown in Fig. 7(c), the wheel-pin contact was subject to a strong impact during the simulation period 0–7 s, and the vibration acceleration of the front walking unit varied by ±400 mm/s^2^. As the simulation continued, the impact gradually decreased, the vibration acceleration of the walking unit gradually stabilized to ±200 mm/s^2^.

### Analysis of frequency-domain response characteristics

**Figure 8:**
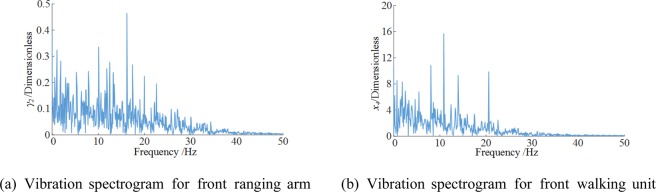
Vibration spectrograms for critical shearer components. (**a**) Vibration spectrogram for front ranging arm (**b**) Vibration spectrogram for front walking unit.

Figure 8 shows the frequency-domain vibration response curves for the front ranging arm and walking unit.

Both the front arm and walking unit vibrated at low frequencies, with their principal frequencies of 17.57 and 11.89 Hz. Notably, both vibration spectra consisted mostly of low frequencies.

### Dynamical behaviors under different settings of shearer operation

**Figure 9:**
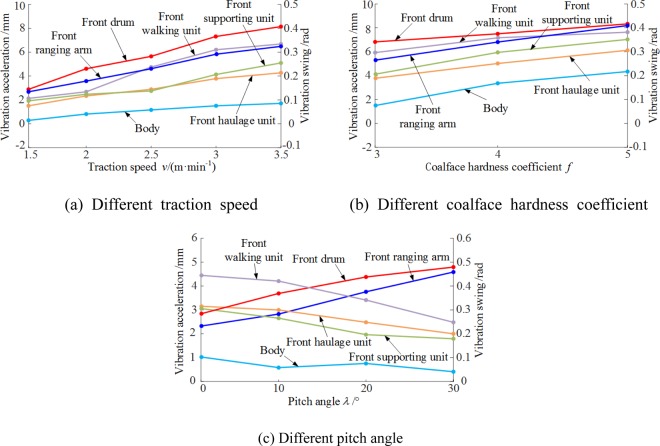
Vibration displacements of critical shearer components with different parameters. (**a**) Different traction speed (**b**) Different coalface hardness coefficient. (**c**) Different pitch angle.

The vibration displacements of critical shearer components under different settings of shearer operating parameters were obtained using the above method combined with a single-variable method. The vibration displacements of the components were averaged to simplify the vibration displacement variation patterns of the components, as shown in Fig. 9.

As shown in Fig. 9(a), with shearer pitch angle kept constant at 0 and coalface hardness coefficient at f = 3, as the traction speed gradually increased from 1.5 to 3 m/min, the vibration-induced displacements/swings of the shearer components increased. The front drum and ranging arm exhibited the same vibration swing, from 0.18–0.41 and from 0.17–0.41 rad, respectively. The vibration displacement of the walking unit exhibited the most significant variations (2.13–5.94 mm). The shearer system changes its traction speed by changing the meshing frequency of the driving wheel and pin rail. Yet, the shearer body, supporting units, and haulage units exhibited smaller variations with the traction speed. These components were compactly coupled and had large inertia.

With the shearer traction speed held constant at 3 m/min and pitch angle at 0, as the coalface hardness coefficient (*f*) increased from 3 to 5, the average vibration displacements of the shearer components increased a little. The difference between the vibration displacements of the supporting and traction units increased, as shown in Fig. 9(b). At *f* = 5, the average vibration swings/displacements of the front drum, ranging arm, and walking unit varied from 0.39–0.42 rad, 0.3–0.41 rad, and 5.94–7.12 mm, respectively. The average vibration swings of the front drum and ranging arm were about equal.

As shown in Fig. 9(c), with the shearer traction speed held constant at 3 m/min and the coalface hardness coefficient at *f* = 3, as the shearer pitch angle increased from 0 to 30°, the vibration swings of the front drum and ranging arm increased gradually from 0.32 to 0.48 rad and from to 0.25 to 0.47 rad, respectively, whereas those of the front haulage, supporting, and walking units and the body decreased gradually. The front walking unit changed the most (4.46–2.64 mm).

## Mechanical Test of Shearer

### Test platform

**Figure 10:**
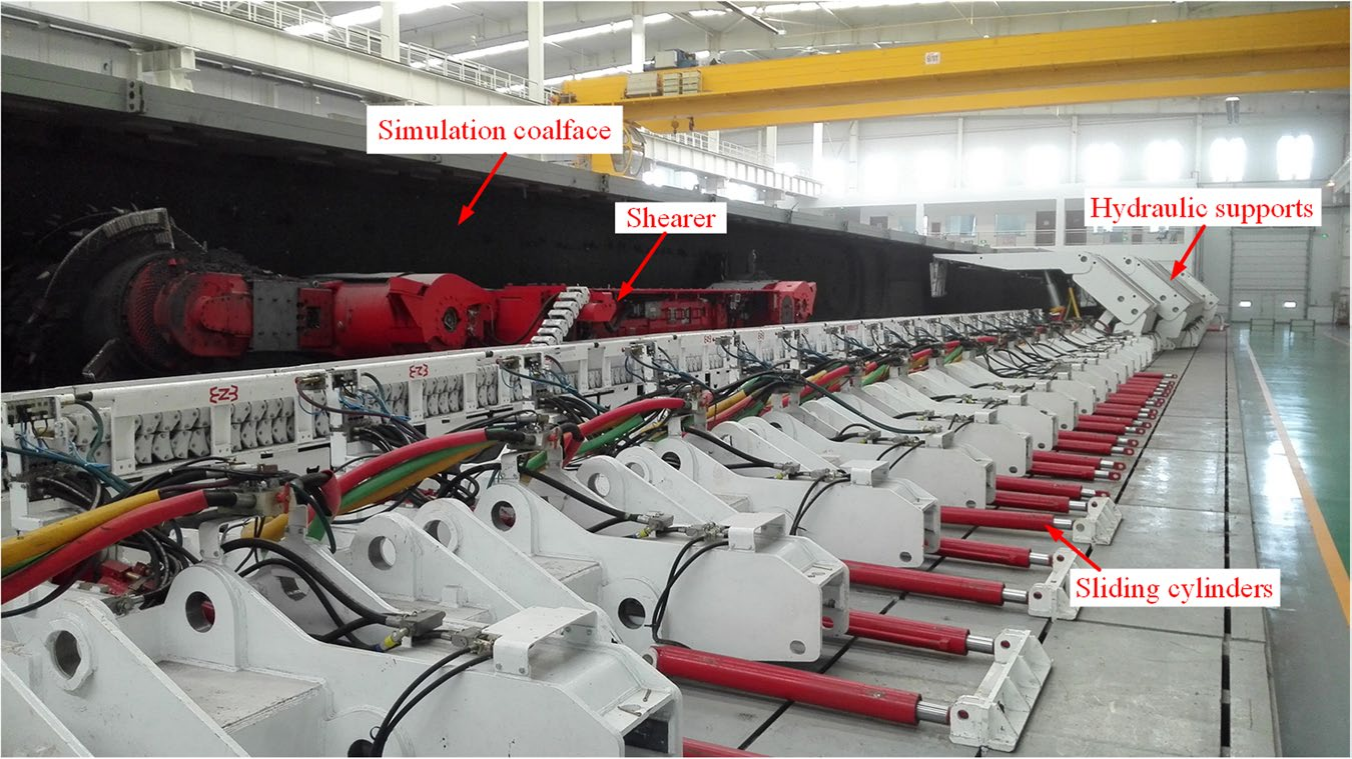
Test platform for shearer mechanical test.

To validate our numerical model, a mechanical test was performed on a shearer using the test platform for mechanical inspection and analysis of fully mechanized coal winning units at the National Energy Mining Equipment Research and Development Center, China Coal Zhangjiakou Coal Mining Machinery Co., Ltd. The platform mainly consisted of the following components: a 1:1 simulated coalface, drum shearer, scraper conveyor, hydraulic supports, coal loader, sliding cylinders, and data acquisition system, as shown in Fig. 10.

The geological and mechanical characteristics of the simulated coalface as compared with real-world coalfaces are critical to the reliability of testing data. Chinese coal reservoirs occur in diversified, complex geological conditions, and underground coal mining involves coal seams of considerably different geological structures and mechanical properties. Thus, the test was intended to simulate only a coalface with typical physical properties and was not intended to be exhaustive. Datong, Shanxi is China’s biggest supplier of high-quality steam coal. The coal seams in this region boast good joint development, low levels of impurities, high caloric value, and high hardness, and are reasonably representative of China’s coal seams. Thus, coal seams in Datong were simulated in the test.

The simulated coalface mainly consisted of coal, which was mixed with cement, water, and water-reducing agent. The coal for the coalface simulation was washed and then crashed into particles measuring 0–50 mm in size. Particles measuring less than 5 mm were used as fine aggregates, while those measuring 5–50 mm were used as coarse aggregates. The cement used was PC32.5, a compound cement with a safe coefficient of cement strength grade of 1.05. The coal and cement were then mixed with appropriate amounts of water and water-reducing agent^27^. The simulation coalface was poured in a layered manner to simulate underground coal seam conditions better. The simulation coalface measured 70 m in length, 4 m in width, and 1.5 m in height. It consisted of two sections of equal lengths but different coalface hardness coefficients (*f*), with the first 35-m section simulated with at *f* = 3 and the second section at *f* = 4.

### Data transmission and display system

**Figure 11:**
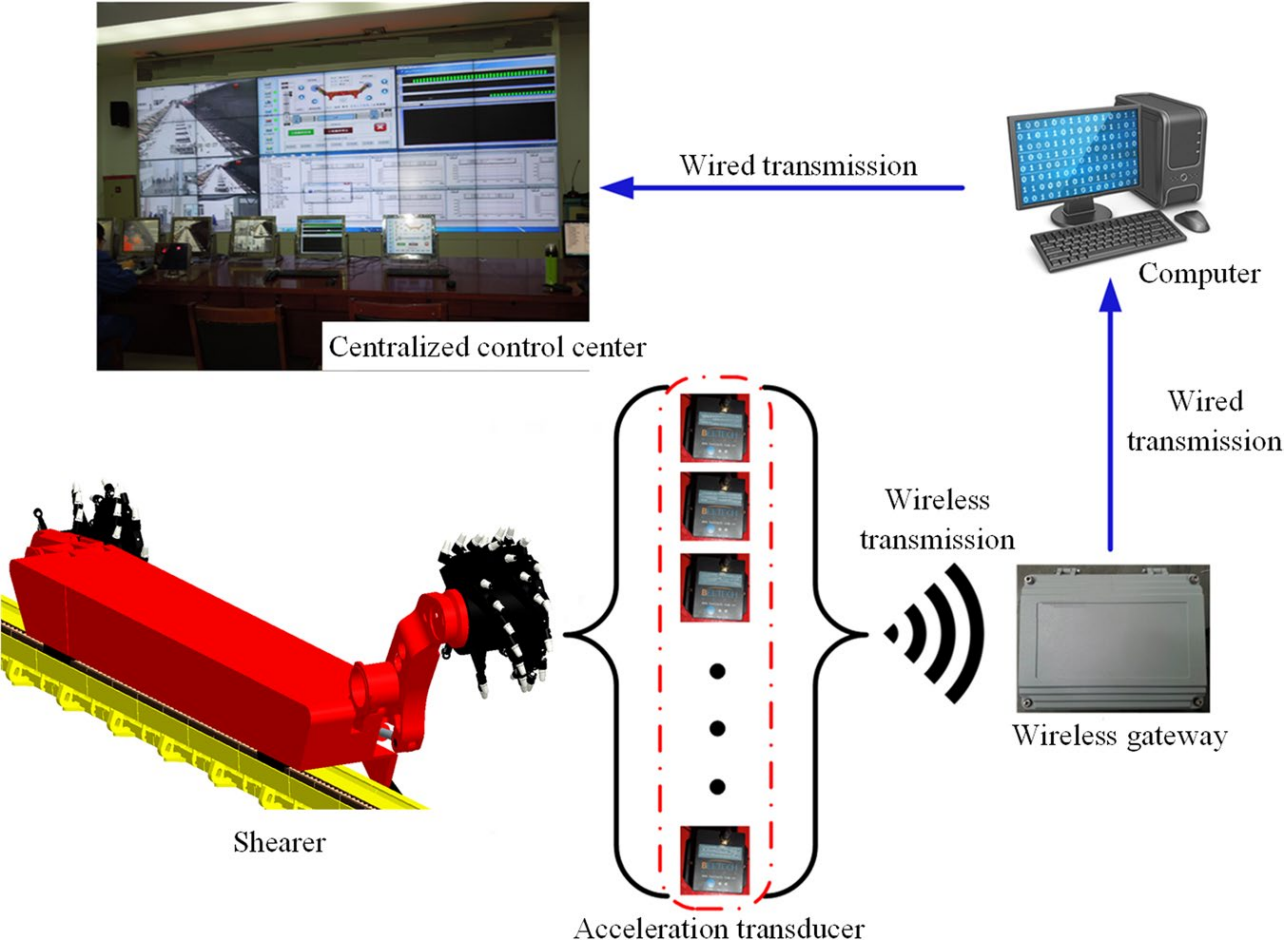
Topological graph of the system for acquiring testing data on shearer critical components.

Because the shearer moves back and forth along the coalface, wired transmission of testing data requires complex wiring and the cables may easily break, resulting in data loss. Complex test wiring is also a safety concern. The test adopted a combination of wired and wireless data transmission to ensure test safety and data collection reliability, as follows: The sensor collected data and transmitted them to a wireless gateway, which then transmitted them in a wired manner to a PC terminal, as shown in Fig. 11. The data signals were amplified and filtered (using median and mean filters) in the PC terminal. The resulting test data was then calibrated and fitted (using Fourier, Gauss, and exponential fitting), with the results transmitted to and graphically displayed in the centralized control center. The data was stored for future analysis.

### Configuration of sensors

**Figure 12:**
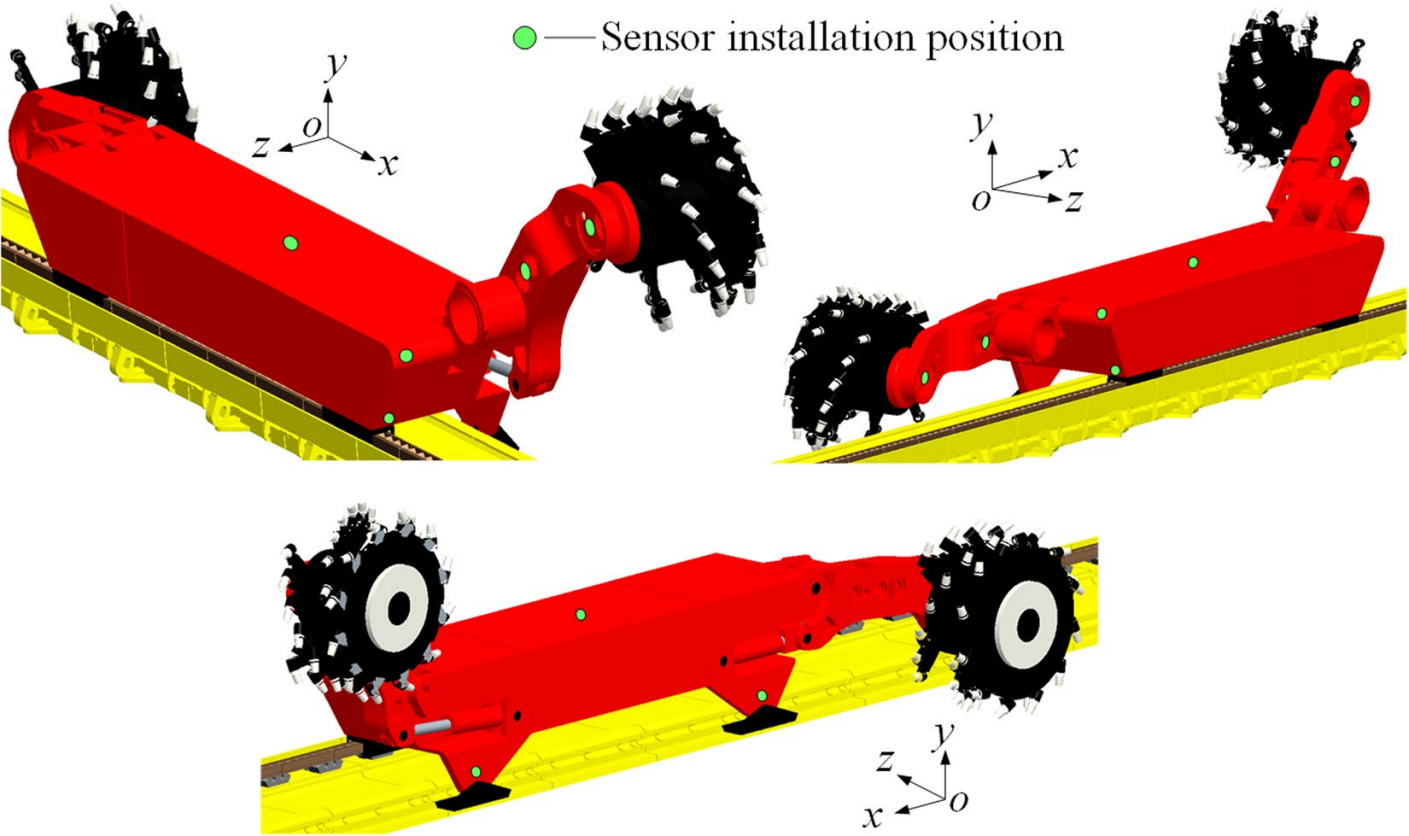
Illustration of transducer configuration.

Wireless acceleration transducers (A301, Beijing Beetech Inc.) were mounted on the following shearer components to test the vibration characteristics: front and rear drums, front and rear ranging arms, front and rear haulage units, front and rear walking units, front and rear supporting units, and shear body, as shown in Fig. 12. The transducers were configured so data could be collected from the transducers accurately, the data collected could reflect the vibration behavior of the shearer under real-world operating conditions, and the mechanical test could be run smoothly, effectively, and safely.

**Figure 13:**
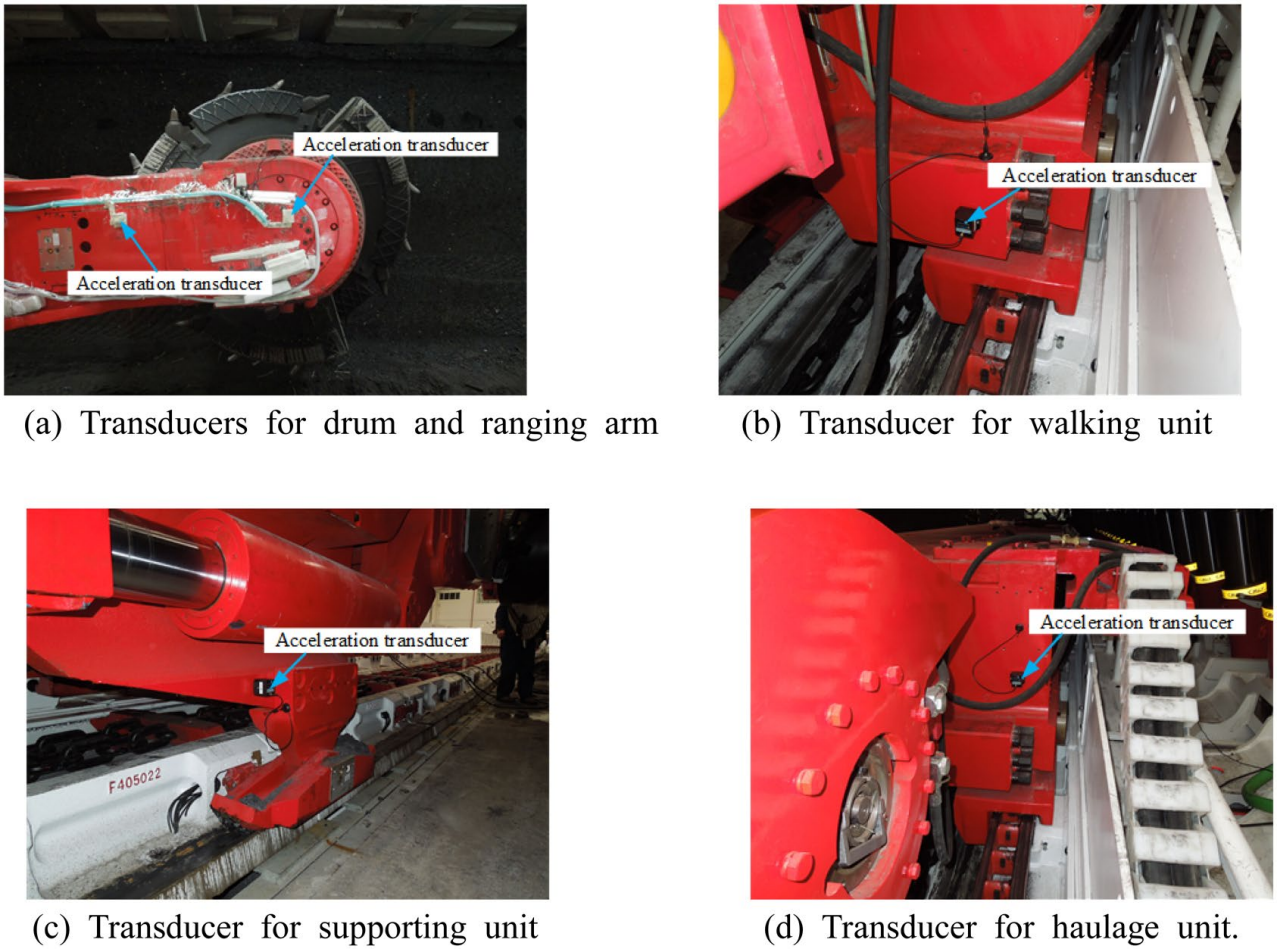
Configuration of transducers. (**a**) Transducers for drum and ranging arm (**b**) Transducer for walking unit. (**c**) Transducer for supporting unit (**d**) Transducer for haulage unit.

The vibration responses of the drums and ranging arms during coal cutting were tested indirectly, because their complex power transmission mechanisms make a direct test close to impossible. Acceleration transducers were mounted as follows:

A transducer for detecting the dynamical responses of the ranging arm was mounted at the front end of the arm, close to its center point of gravity and not subject to the impact of coal falling from coalface cutting, and another transducer was mounted at the gravity center of the ranging arm and properly encapsulated to test the vibration responses of the ranging arm, as shown in Fig. 13(a).

Transducers of the walking and supporting units were configured so they could not drop nor be impacted by falling coal, as the shearer moved relative to the scraper conveyor. The transducer for the vibration responses of the walking unit was mounted near its gravity center, that neither the transducer nor its transmitting antenna could be affected by falling coal and the spill plate and pin rail of the scraper conveyor, as shown in Fig. 13(b). The transducer for the vibration responses of the supporting unit was mounted ear its gravity center below the lift cylinder on the shearer body side, in similar fashion to protect the transducer from falling coal, as shown in Fig. 13(c).

The transducer for the vibration responses of the haulage unit was mounted near its gravity center on the side vertical to the walking direction, again protected from falling coal, as shown in Fig. 13(d). Lastly, the transducer for vibration responses of the shearer body was mounted near its gravity center on its top.

### Comparison of numerical and testing results

**Figure 14:**
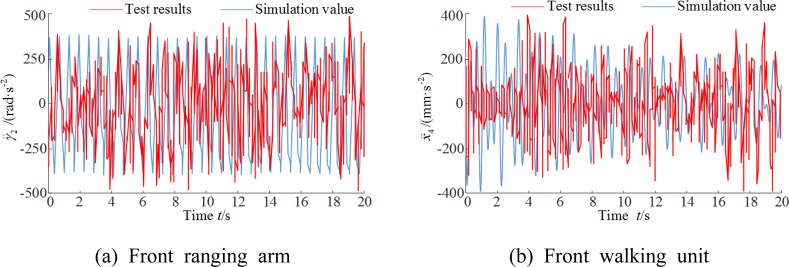
Vibration accelerations. (**a**) Front ranging arm (**b**) Front walking unit.

**Figure 15:**
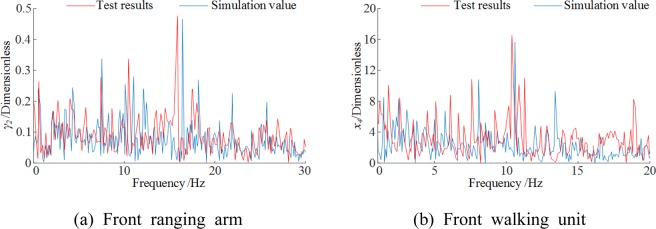
Vibration frequency spectrograms. (**a**) Front ranging arm (**b**) Front walking unit.

The vibration accelerations and frequency-domain responses of the front ranging arm and walking unit from simulation and test were compared. The operating parameters were set at a 3 m/min traction speed, the coalface hardness coefficient of *f* = 3 and a shearer pitch angle of 0. Figure 14 compares the vibration acceleration curves and Fig. 15 the spectrograms.

The vibration acceleration responses of the ranging arm and walking unit obtained from the simulation and test were largely consistent in frequency and fluctuation. The responses from the test exhibited temporary large fluctuations, possibly due to factors including the uneven floor the shearer operated on. The principal vibration frequencies of the ranging arm and walking unit from the simulation only slightly deviated from those of the test. The test spectrograms contain more vibration frequencies than the simulation. One explanation is the complex gear transmission mechanisms inside the arm and walking unit which makes them sensitive to the hydraulic and electrical systems inside the shearer body. Compared to the test results, the simulation had small errors below 10%, as shown in Table 6.

**Table 6:** Comparison of numerical and testing results.

	Front ranging arm		Front walking unit	
	Vibration swing rad/s^2^	Frequency/Hz	Vibration acceleration mm/s^2^	Frequency/Hz
Simulation value	−0.187	17.57	−0.171	11.89
Test results	−0.203	16.74	−0.185	11.23
Relative error	7.82%	4.96%	7.57%	5.88%

The vibration displacements of shearer components under different operating conditions from the simulation were compared with those in the test, as shown in Tables 7 and 8. Note Table 8 compares only the vibration behaviors of shearer components in different directions obtained from the simulation and experiment at *f* = 4, because the simulation and experimental results for *f* = 3 are already included in Table 6. Under all the different settings, the vibration displacements of shearer components obtained from the simulation are larger than those of the experiment, but with errors of approximately 10%.

**Table 7:** Vibration displacements of critical shearer components at different traction speeds obtained from the simulation and test.

*v* m/min		1.5	Relative error	2	Relative error	2.5	Relative error	3	Relative error	3.5	Relative error
Haulage unit/mm	Simulation value	1.87	3.61%	2.11	8.66%	2.21	7.53%	3.14	3.98%	3.47	1.14%
	Test results	1.94		2.31		2.39		3.27		3.51	
Walking unit/mm	Simulation value	2.13	7.93%	2.27	7.72%	4.26	8.97%	5.83	3.00%	5.94	6.16%
	Test results	2.30		2.46		4.68		6.01		6.33	
Supporting unit/mm	Simulation value	1.97	9.63%	2.16	7.69%	2.14	9.70%	3.25	4.97%	4.22	3.43%
	Test results	2.18		2.34		2.37		3.42		4.37	
Body/mm	Simulation value	0.24	4.00%	0.35	10.26%	0.38	5.00%	1.07	3.60%	1.26	8.70%
	Test results	0.25		0.39		0.40		1.11		1.38

**Table 8:** Vibration displacements of critical shearer components at different coalface hardness obtained from the simulation and test.

Coalface hardness coefficient *f* = 4	Simulation value	Test results	Relative error
Front haulage unit /mm	4.51	4.72	4.45%
Front walking unit /mm	7.12	7.87	9.53%
Front supporting unit /mm	5.42	5.96	9.06%
Body /mm	3.31	3.46	4.34%

The errors may be explained by items not considered in the simulation:

1. Difference in the heights of adjacent middle troughs
2. Effect of the vibration of the gear transmission systems of the coal cutting and walking units on the vibration of the whole shearer
3. Effect of the coal gangue under the sliding shoe on the vibration of the whole shearer
4. Deviation of the actual dimensions from the design, nor the effect of the shearer assembly error on the design dimensions
5. Effect of the vibration of the shearer electrical and hydraulic systems on the vibration of the whole shearer.
6. Experimental and data processing errors.

## Conclusions

A numerical model of the dynamical behavior of a shearer at real-world operating conditions was developed. The couplings between critical shearer components and the contact between the shearer and scraper conveyor were considered. The tangential rigidity of the coupling between the sliding shoe and middle trough was simulated using three-dimensional fractal theory. The rigidity of the gapped contact between the driving wheel and pin rail was simulated using Hertz contact theory, and the lift/supporting unit and the contact between the shearer body and traction unit were characterized using Hooke’s law. On this basis, a nonlinear dynamic model of the shearer traction-swing coupling with 13 degrees of freedom using vibration mechanics and multi-body dynamics was established. The model was tested with an experimentally corrected drum load as the external excitation. In summary:

1. A coefficient that helps correct drum load against traction speed was proposed to determine the load on the shearer drum. It was determined using mechanical test results. The errors of the corrected drum loads in three directions were small (0.77%, 2.67%, and 1.59%), which confirms the corrected drum loads.
2. Dynamical responses of critical shearer components were obtained using the numerical model and the corrected drum loads. At a shearer traction speed of 3 m/min, pitch angle of 0, and coalface hardness coefficient of *f* = 3, the front section of the shearer exhibited strong vibration responses. The vibration displacements/swings of the front drum, ranging arm, and walking unit varied greatly from −0.4–0.8 rad, −0.1–0.5 rad, and by ±12 mm, respectively. The vibration acceleration of the front ranging arm and walking unit varied by ±380 rad/s^2^ and ±200 cmm/s^2^, respectively. The vibrations of these two components were chaotic, with their principal vibration frequencies equal to 17.57 and 11.89 Hz, respectively.
3. The average vibration displacements of critical shearer components at different shearer settings were obtained using a single variable method. The vibration displacements/swings of shearer components increased with the traction speed increase to 3 m/min. The front drum, ranging arm, and walking unit exhibited large displacements/swings. The vibration displacements/swings of the front drum, ranging arm, and walking unit varied from 0.18–0.41 rad, 0.17–0.41 rad, and 2.13–5.94 mm, respectively. The average vibration displacements/swings of shearer components increased slowly when the coalface hardness coefficient (*f*) increased from 3 to 5. The displacements/swings of the front drum, ranging arm, and walking unit ranged from 0.39–0.42 rad, 0.30–0.41 rad, and 5.94–7.12 mm, respectively. As the shearer pitch angle increased from 0–30°, the vibration displacements/swings of the front drum, ranging arm, and walking unit changed sharply. The vibration swings of the drum and ranging arm increased along with the pitch angle that changed from 0.32–0.48 and from 0.25–0.47 rad. The vibration displacement of the front walking unit decreased with an increase in the pitch angle, and changed from 4.46–2.64 mm.
4. A shearer mechanical test was performed on a test platform for fully mechanized coal face to validate the model. The simulation results and the testing results at different shearer operating conditions were compared. The relative errors of the numerical model were small, all below 10%, thereby confirming the accuracy of the numerical model. This means our model can reasonably well describe and predict the dynamical behavior of the shearer traction-swing coupling. The remaining errors originated in not considering shearer hydraulic, electrical, and gear transmission systems on the whole shearer in the numerical simulation.

The research results provide theoretical basis for the structure optimization and process parameter optimization of the shearer.

The research results can guide the optimal control of the operating parameters of the shearer under different working conditions and provide a theoretical basis for the reliability of the shearer and the fatigue life prediction of key components.
